# Is it a match? a novel method of evaluating medical school success

**DOI:** 10.1080/10872981.2018.1432231

**Published:** 2018-02-13

**Authors:** Leslie L. Chang, Alisa Nagler, Mariah Rudd, Colleen O’Connor Grochowski, Edward G. Buckley, Saumil M. Chudgar, Deborah L. Engle

**Affiliations:** a Duke University School of Medicine, Durham, NC, USA; b Division of Education, American College of Surgeons, Chicago, IL, USA; c Office of Continuing Professional Development, Virginia Tech Carilion School of Medicine, Roanoke, VA, USA; d Office of Curricular Affairs, Duke University School of Medicine, Durham, NC, USA; e Department of Ophthalmology, Duke University School of Medicine, Durham, NC, USA; f Department of Medicine, Duke University School of Medicine, Durham, NC, USA

**Keywords:** Match, undergraduate medical education, program evaluation, residency, specialty, reputation

## Abstract

**Background:** Medical education program evaluation allows for curricular improvements to both Undergraduate (UME) and Graduate Medical Education (GME). UME programs are left with little more than match rates and self-report to evaluate success of graduates in The Match.

**Objective:** This manuscript shares a novel method of program evaluation through a systematic assessment of Match outcomes.

**Design:** Surveys were developed and distributed to Program Training Directors (PTDs) at our institution to classify residency programs into which our UME graduates matched using an ordinal response scale and open-ended responses. Outcomes-based measures for UME graduates were collected and analyzed. The relationship between PTD survey data and UME graduates’ outcomes were explored. Open-ended response data were qualitatively analyzed using iterative cycles of coding and identifying themes.

**Results:** The PTD survey response rate was 100%. 71% of our graduates matched to programs ranked as ‘elite’ (36%) or ‘top’ (35%) tier. The mean total number of ‘Honors’ grades achieved by UME graduates was 2.6. Data showed that graduates entering elite and top GME programs did not consistently earn Honors in their associated clerkships. A positive correlation was identified between USMLE Step 1 score, number of honors, and residency program rankings for a majority of the programs. Qualitative analysis identified research, faculty, and clinical exposure as necessary characteristics of ‘elite’ programs:. Factors considered by PTDs in the rating of programs included reputation, faculty, research, national presence and quality of graduates.

**Conclusions:** This study describes a novel outcomes-based method of evaluating the success of UME programs. Results provided useful feedback about the quality of our UME program and its ability to produce graduates who match in highly-regarded GME programs. The findings from this study can benefit Clerkship Directors, Student Affairs and Curriculam Deans, and residency PTDs as they help students determine their competitiveness forspecialties and specific residency programs.

## Introduction

A cycle of frequent medical education program evaluation followed by quality improvement initiatives is crucial to educate future physicians effectively in the constantly changing world of healthcare. Identification of educational program deficiencies through regular comprehensive evaluation of programs allows for curricular improvements to be made in both Undergraduate Medical Education (UME) and Graduate Medical Education (GME).

Aspects of program evaluation that are routinely examined in UME [1,2] include learner outcomes (such as competencies achieved), clerkship grades, board examination scores, and results from the annual National Resident Matching Program® (NRMP® or The Match®). Match results are critical for UME programs, as the success of graduates helps shape the school’s reputation and the caliber of future students it attracts.

Within UME program evaluation, the definition of a successful Match cycle may be wide-ranging. The Liaison Committee for Medical Education (LCME) requires that UME programs report Match rates as an outcome indicator of the educational program. UME programs may also query their graduates about whether they matched to their first, second, or third choice of residency programs. This data is not officially released by Electronic Residency Application System® (ERAS®), therefore UME programs must rely on student self-report. In addition, UME programs may track the GME institutions to which their graduates match. Success may be defined as a majority of graduates matching into residency programs that the UME institution perceives as being of high quality.

The definition of high-quality residency programs is not well-articulated or standardized on a national level. Little is published on the topic of residency program rankings or comparisons despite a clear demand from prospective trainees and other stakeholders. Unlike the rankings of the best hospitals and best medical colleges that have existed in *U.S. News and Reports* (*USNWR*) listings since the 1990s, there has not been a similar ranking system for GME residency programs.

This changed in 2014, when Doximity, in collaboration with *USNWR*, released rankings of residency training programs by specialty. Doximity is a free, HIPAA-compliant, online network of physicians that facilitates social and professional connections and provides educational resources. Doximity rankings are based on a combination of resident survey data, reputation of program survey data, and research output data [3–5]. As such, Doximity rankings have begun to fill the void of ranking GME programs.

However, there has been some criticism of the utility of Doximity, as given rankings are based on reputation as defined by survey-eligible physicians or based on research productivity [6,7]. Furthermore, the quality of residency programs can be expressed in many outcome measurements and a variety of factors may be required to fully capture program quality.

Recently, researchers attempted to rank residency programs within a single specialty (general surgery) based on publicly available program outcome measures [8]. In this study, all 254 general surgery residency programs were rank-ordered using board pass rates and the prevalence of alumni publications. Seventeen programs that were ranked in the top 30 according to reputation were also ranked in the top 30 based on outcomes measures. Therefore, only a moderate association was found between programs ranked by these outcomes measures and those ranked according to reputation, suggesting that multiple quantifiable indicators should be used to measure program quality and not reputation and/or research output alone.

In the absence of outcome studies like the research cited above for every residency program/specialty outside of general surgery, along with the limitation of the current Doximity ranking system, UME programs are left with little more than match rates and self-report to evaluate the success of their graduates in The Match.

Prompted by this gap, a research team comprising members from UME and GME programs sought to investigate a process to enrich our program evaluation efforts through a more systematic assessment of our own Match outcomes. Residency PTDs have the responsibility of knowing the caliber and competitiveness of programs in their respective fields. Therefore, we specifically aimed to explore factors that residency PTDs consider in ranking GME programs and the relationships between quantitative measures of student achievement (such as clerkship grades) and residency PTD rankings. This article serves to share the utility of this novel method of program evaluation and the process we used, so that other institutions may adopt this innovation and learn from their own data. This article also seeks to contribute to the limited literature on program evaluation as it relates to The Match.

## Methods

We identified each of the core GME residency programs (n = 20) that our UME graduates (n = 217) matched to between the years of 2011 and 2013. We then created specialty-specific surveys, wherein we asked the residency PTDs (n = 20) at our institution to use their professional judgment to classify the residency programs listed, according to this ordinal response scale, ‘lower,’ ‘middle,’ ‘top,’ or ‘elite’ tier programs. We also asked PTDs to respond to the following open-ended questions: ‘What factors did you consider when rating these programs?’ and ‘What qualities or factors need to be present in order for a GME training program to be considered elite?’

## Data sources

We sent a specialty-specific survey to each PTD of our core GME programs that asked them to rank individual residency programs as described above. We asked them to consider each program in relation to all institutions nationwide, not just the programs listed. From this data, we sought to answer:

- How do our own residency PTDs rank the GME programs into which our graduates match?
- What factors did PTDs consider when rating these programs?
- What qualities or factors need to be present in order for a GME training program to be considered ‘elite’ according to PTDs?

In addition, we collected outcomes-based measures for each UME graduate related to two factors that residency programs often use to screen applicants: clerkship grades and board scores. Transcript data was analyzed for the number of Honors, High Pass, and Pass grades that each graduate achieved during their respective clerkship year which consist of six core clerkships: Family Medicine, Internal Medicine, Obstetrics and Gynecology, Pediatrics, Psychiatry, and Surgery. We tallied the number of Honors grades achieved by each student during the clerkship year. Scores on the USA Medical Licensing Exam (USMLE) Step 1 were also collected and analyzed. Using this data along with PTD survey data described above, we sought to answer:

- Is there a relationship between the PTD rankings and the number of Honors grades that students earn during their clerkship year?
- Is there a relationship between the PTD rankings and the student scores on Step 1?
- What is the relationship between the PTD ranking of GME Programs and the grade distribution of the associated clerkship?

## Analysis

We used both qualitative and quantitative approaches to data analysis. Descriptive statistics were calculated for residency program rankings, clerkship grades, and USMLE Step 1 scores. In order to examine whether the total number of clerkship Honors and Step 1 scores differed between the program rankings, we conducted Kruskal Wallis tests given a lack of normal distribution as ascertained by Shapiro-Wilk tests. Significance level was set at p </ = 0.05.

The open-ended response text data was qualitatively analyzed using iterative cycles of coding and identifying themes. Two authors (AN and MR) worked independently to review the data continuously until common themes emerged and saturation was reached. Authors compared their findings and any discrepancies were discussed until there was a shared set of themes. Deviations from such themes or patterns were also noted.

## Results

### How do our own PTDs rank the GME programs into which our graduates match?

All PTDs who received the survey responded (response rate = 100%). According to survey results of our PTD rankings, 71% of graduates matched to programs ranked as ‘elite’ (36%) or ‘top’ (35%) tier. The mean total number of Honors grades achieved by UME graduates across all clerkship courses was 2.6 (range = 0 to 6) (Table 1). The mean USMLE Step 1 score was above the current (December 2017) national mean of 228.

**Table 1.** Residency program rank and UME graduate characteristics.

Residency Program Rank	Total Number of UME graduates who matched to residency program Frequency of Total (%)	Graduates’ Mean Total Number of ‘Honors’ grades across all clerkships (range = 0 to 6)	Graduates’ Mean USMLE Step 1 score Above or Below Current National Mean of 228
Elite	78 (36%)	3.7	Above
Top	75 (35%)	3.1	Above
Middle	47 (22%)	2.1	Above
Lower	16 (7%)	1.3	Below
Tally	217 (100%)	Mean = 2.6	Mean = Above

### What factors did PTDs consider when rating these programs?

Thematic response to this open-ended question included: reputation, faculty caliber, research, national presence and quality of graduates. Other factors included: length of time program has been in existence, size of program, retention of program director, resident awards and accolades at national meetings, and competitiveness of incoming classes.

### What qualities or factors need to be present in order for a GME training program to be considered elite?

In order for a GME training program to be considered elite, the following themes emerged from responses as necessary qualities or factors: research, faculty, and clinical exposure (Table 2).

**Table 2.** Representative responses to ‘What qualities or factors need to be present in order for a GME training program to be considered elite?’.

Theme	Individual Responses
Research	*Have a formal research requirement for the residents* *Research commitment by chairman and program director to value the resident’s time in pursuing a topic at depth during training* *Significant and consistent contributions to the literature* *Residents with publications and/or who win national awards*
Faculty	*National prominence of multiple faculty members* *Solid diverse faculty who are experienced* *Internationally recognized faculty*
Clinical Exposure	*No deficiencies in patient care opportunities* *Clinical exposure and successful clinical training first and foremost (evidenced by excellent board certification rates, graduates who are sought out, etc.)* *Excellent clinical reputation, breadth of clinical experience and research*

### Is there a relationship between PTD rankings and the number of honors grades that students earn during their clerkship year? is there a relationship between the PTD rankings and the student scores on step 1?

Table 1 also shows that as the rank of the programs increased from lower tier to elite, the mean number of Honors grades and mean Step 1 scores also increased. Beyond this clear relationship, we analyzed whether these differences in Honors grades were statistically significant across program rankings (lower vs. middle vs. top vs. elite). Results of the Kruskal Wallis test showed there was a significant difference (H (3) = 31.9, *P *< 0.0001). We also analyzed whether there was a difference in mean Step 1 scores across program rankings. Results of the Kruskal Wallis test showed there was a significant difference (H (3) = 14.3, *P *= 0.0025) here as well.

While Table 1 illustrates aggregate data for all GME programs in aggregate, Table 3 shows descriptive statistics at the individual program level. A mean of 3 Honors grades and a mean Step 1 score above the national average yielded a successful match into the first 10 programs listed in Table 3, which are specialties widely-known as more competitive.

**Table 3.** Individual residency program rank and UME graduate characteristics.

Core Residency Program	Total Number of UME graduates who matched to residency program Frequency of Total (%)	Graduates’ Mean Total Number of ‘Honors’ grades across all clerkships (range = 0 to 6)	Graduates’ Mean USMLE Step 1 score Above or Below Current National Mean of 228
Specialty A – medical	7 (3%)	4.3	Above
Specialty B – surgical	3 (1%)	4.3	Above
Specialty C – surgical	15 (7%)	4.1	Above
Specialty D – surgical	4 (2%)	4.0	Above
Specialty E – medical	10 (5%)	3.8	Above
Specialty F – medical	3 (1%)	3.7	Above
Specialty G – surgical	13 (6%)	3.7	Above
Specialty H – medical	50 (23%)	3.1	Above
Specialty I – surgical	5 (2%)	3.0	Above
Specialty J – surgical	4 (2%)	3.0	Above
Specialty K – medical	6 (3%)	2.9	Below
Specialty L – surgical	10 (5%)	2.6	Below
Specialty M – medical	14 (6%)	2.5	Above
Specialty N – medical	8 (3%)	2.4	Above
Specialty O – medical	14 (6%)	2.4	Below
Specialty P – medical	22 (10%)	2.4	Below
Specialty Q – surgical	9 (4%)	2.1	Below
Specialty R – medical	12 (5%)	2.0	Below
Specialty S – surgical	5 (2%)	2.0	Above
Specialty T – medical	3 (1%)	1.7	Below
	Total = 217 (100%)	Mean = 2.6	Mean = Above

The specialties designated here as ‘medical’ include: anesthesiology, Dermatology, Diagnostic Radiology, Emergency Medicine, Family Medicine, Internal Medicine, Medicine/Pediatrics, Neurology, Pediatrics, Psychiatry, and Radiation Oncology.

The specialties designated here as ‘surgical’ include: General Surgery, Integrated Plastic Surgery, Neurosurgery, Obstetrics/Gynecology, Ophthalmology, Orthopedic Surgery, Otolaryngology, Plastic Surgery and Urology.

As a next step, we focused our analysis to only the number of graduates who matched into individual GME programs ranked as elite, along with the associated tally of Honors grades and Step 1 scores. Kruskal-Wallis tests across the elite individual GME programs were performed, but showed no significant difference for total number of Honors grades or for Step 1 scores. Similar focused analyses were completed for individual programs ranked as top, as middle and as lower tier. Kruskal-Wallis tests across the individual programs at each ranking showed no significant differences for total number of Honors grades or Step 1 scores. Data are not shown as the numbers are small in some cases and student identity could be inferred.

### What is the relationship between the PTD ranking of GME programs and the grade distribution of the associated clerkship?

**Figure 1.**
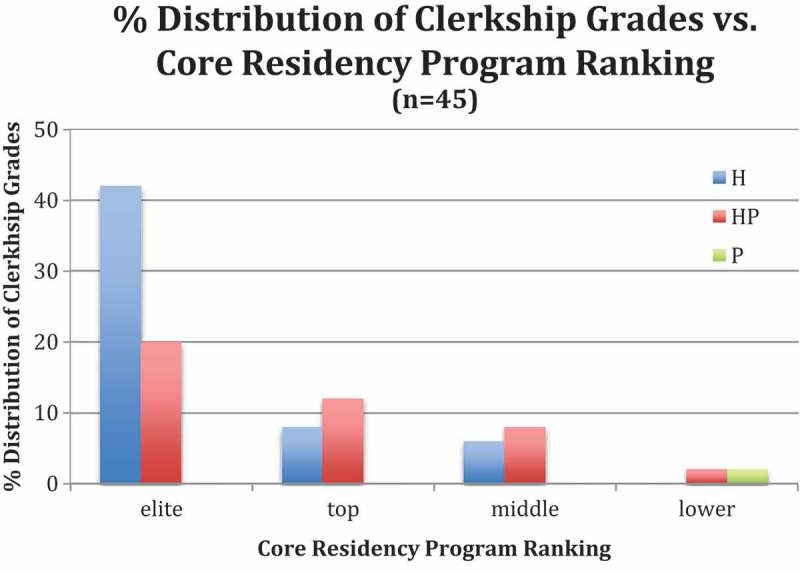
Representative Clerkship Data. Distribution of grades in clerkship associated with core residency program ranking.

We analyzed the relationship between the program ranking and the distribution of summative grades that our graduates earned in the associated clerkship (i.e. Internal Medicine program ranking vs. grade distribution of Internal Medicine clerkship). Generally, the data revealed that graduates entering elite and top GME programs did not consistently earn Honors in the associated clerkships. For surgical subspecialties, however, the more Honors earned, the higher the ratings of the GME programs that the student matched into (Figure 1). As described earlier, actual data are not shown because of the low number in some cases.

## Discussion

We describe a novel, outcomes-based method of evaluating the success of UME programs by using an internally administered survey of PTDs. Furthermore, we utilize a new approach to determine quality of GME programs where our graduating students match; specifically, PTDs familiar with nation-wide programs in their specialty evaluated and ranked them into tiers. Apart from Doximity, little work has been done previously to rank core GME programs in order to allow schools to make comparisons between programs.

Quantitative results show that nearly three-quarters of our graduates matched in elite or top residency programs as assessed by specialty-specific PTDs. From a program evaluation perspective, we believe this demonstrates that we have a strong educational program and that we are producing graduates who are desired by well-respected programs. Only 7% of our graduates matched at programs considered by our specialty-specific PTDs to be lower tier programs. Again, these findings seem to support the strength of our educational program. However, we are not able to determine from these findings if this 7% of graduates ranked their residency programs on the basis of characteristics independent of these programs (*e.g*., geographical location or proximity to family).

We also showed that when GME programs were aggregated according to rank, clerkship grades and step scores were statistically different among the rankings. In other words, learners with more honors grades and higher Step 1 scores had matched into higher ranked programs. This finding is consistent with the 2017 Gauer and Jackson study [9], which found that Step 1 scores are significantly associated with specialty match.

Qualitative analysis revealed that reputation was the most prevalent theme in the response to what factors were considered when ranking the programs. A study conducted by Wilson *et al*. found that reputation alone does not fully capture the quality of the program, but that the combination of reputation or peer review combined with quantifiable indicators may be more meaningful [8]. We agree and thus our study used quantitative objective data (clerkship grades and Step 1 scores), which supported the subjective rankings.

The other themes identified from the qualitative data (faculty, research, national presence and quality of graduates) are more quantifiable and may be meaningful variables to measure in evaluating the GME programs into which our graduates match. The more unique responses or deviations (such as tenure of the program director and national awards) illustrate other variables that program directors report are important in determining program quality and also are more quantifiable. This list of additional variables supports our *a priori* assumption that program directors had knowledge about the GME programs in their field that they were ranking and that it was not done just based on a hunch.

Research, faculty, and clinical exposure were themes identified from the question, ‘[w]hat qualities or factors need to be present in order for a GME training program to be considered elite’ but came up minimally if at all for the question about what factors responders were considering when rating the programs. These factors expand beyond the more traditional residency training and ACGME competencies such as patient care and medical knowledge [10]. This suggests that elite programs may include experiences and curriculum which go beyond the minimum required by ACGME and are aligned with the goals of our own curriculum which includes very early clinical exposure and a year-long focus on research.

In addition to providing program evaluation data, the findings from this study can also benefit Clerkship Directors, Student Affairs Deans, and PTDs as they help students determine their competitiveness for both specialties and specific residency programs within those. This advice is often founded in years of experiences and gestalt, as many residency programs may not publish specific guidelines for how they determine which students they will interview or accept to their program. Some of these drivers have contributed to application inflation, with students applying to an increasing total number of programs, causing PTDs to have even more applications to review [11]. A periodic analysis like the one conducted in this study may help advisors in guiding their students to apply to a more streamlined and appropriate number of programs. Finding a method for students to determine their competitiveness can help them focus their applications to programs that are the best fit for them. This particular study revealed that PTDs may take a more holistic approach to reviewing applicants than just looking at numbers. For example, of the graduates in this study who matched at elite programs, greater than half had a grade of Honors in fewer than four out of six clerkships, and a handful had fewer than three Honors. This trend in clerkship grades continued for students who matched at top programs: greater than half the student matching in top programs had fewer than three Honors in the six clerkships. These findings highlight that it is still possible to match to a program considered by our program directors to be an ‘elite’ or ‘top’ program even if the student did not achieve a grade of Honors in all or most clerkships.

Our study did have some limitations. A reputation-based survey will fail to adequately account for students who prioritize other factors over reputation of residency program for various reasons. For example, students may be limited geographically to particular areas of the USA, thus restricting their access to elite institutions. Additionally, some students prefer to match at a program for a select track, area of expertise, anticipated quality of life, or specialty culture. The proportion of students with these types of considerations is difficult to account for in this retrospective study. Another limitation is the small number of individuals located at one institution participating in the specialty-specific surveys. By design, we identified the PTD of each specialty as the ideal individual to complete the survey, likely having knowledge about and insight of the other programs in their specialty across the country. Yet, we acknowledge that conducting a national survey of PTDs in the future might improve the value of this method of evaluation.

## Conclusion

Medical schools seek ways to evaluate their programs in order to do continuous improvement. One method of program evaluation is to look at our match rate and to which institutions our graduates go. We incorporated an internal ranking of GME programs and found that the vast majority (71%) of our students matched at ‘elite’ and ‘top’ programs. PTD rankings were generally supported by the quantitative data we analyzed regarding medical student success (clerkship grades and Step 1 scores), though we did find that achieving Honors in all or even most clerkships was not a necessary prerequisite to matching at an ‘elite’ or ‘top’ program. This study provided useful feedback about the quality of our UME educational program and its ability to produce graduates who can match in highly-regarded GME programs.
